# Introduction to the special issue: Health and social drivers in the criminal justice system

**DOI:** 10.1017/cts.2022.462

**Published:** 2023-03-02

**Authors:** Nickolas Zaller, Michele Staton, Megha Ramaswamy

**Affiliations:** 1 Department of Health Behavior and Health education, University of Arkansas for Medical Sciences Fay W. Boozman College of Public Health, Little Rock, AR, USA; 2 Department of Behavioral Science, College of Medicine, University of Kentucky, Lexington, KY, USA; 3 Department of Population Health, School of Medicine, Kansas University, Kansas City, KS, USA

The USA has the highest incarceration rate in the word, comprising ∼5% of the world’s population, but accounting for ∼25% of the world’s incarcerated population [1]. This means that on any given day, nearly two million individuals are incarcerated in the USA. Yet, not all Americans have the same risk for incarceration. Black Americans are, on average, more likely to be targeted every step along the criminal justice (CJ) continuum including interactions with law enforcement, incarceration, and community supervision [2]. And, while factors contributing to the enormous racial disparities in incarceration are varied and complex, the end result is clear: if current rates of imprisonment persist, one in three Black men born today will be incarcerated during his lifetime [3].

*There is clear and compelling evidence that incarcerated individuals are disproportionately afflicted by chronic physical and mental illness.* For example, individuals with a history of incarceration are more likely to have asthma, diabetes, and incident hypertension relative to the general population [4–5]. Substance use rates among incarcerated persons exceed 80%, and nearly 50% meet diagnostic criteria for co-occurring mental illness and substance use disorder upon intake [6]. Incarceration can also exacerbate or cause new onset of diseases and disorders through infection exposure, inadequate medical care, and traumatic stress [7]. While some evidence-based clinical and/or care management strategies for patients returning from correctional facilities to the community have been developed [8–10], these are insufficient to address the growing health disparities among CJ-involved populations. There is therefore an urgent need to engage researchers across a broad range of disciplines to conduct clinical and translational research to advance our understanding of how to best ameliorate the adverse consequences of CJ involvement.

The Clinical and Translational Science Awards (CTSA) program represents a robust research infrastructure “designed to develop innovative solutions that will improve the efficiency, quality and impact of the process for turning observations in the laboratory, clinic, and community into interventions that improve the health of individuals and the public” [11]. More than 50 CTSAs are currently funded throughout the USA, each with the necessary research capacity to conduct the science that will improve clinical care and population health. However, this vast network has not previously prioritized research activities among individuals and communities most impacted by incarceration. The articles included in this special issue of the journal collectively represent some of the ways in which CTSAs leverage their considerable resources to support research aimed at improving the health and well-being of CJ-involved populations, as well as ethnical conduct of research, community engagement, and the importance of academic–correctional partnerships. We hope this special issue accomplishes the following:

1. Raises awareness about the health disparities and inequities faced by groups targeted by the criminal legal system continuum;
2. Increases motivation of clinical and translational science researchers to become engaged in this research;
3. Provides best practice strategies that researchers can employ to conduct this research (see examples here www.kumc.edu/cjtracs).
